# Clinical Significance of Variants in the *TTN* Gene in a Large Cohort of Patients With Sporadic Dilated Cardiomyopathy

**DOI:** 10.3389/fcvm.2021.657689

**Published:** 2021-04-30

**Authors:** Lei Xiao, Chenze Li, Yang Sun, Yanghui Chen, Haoran Wei, Dong Hu, Ting Yu, Xianqing Li, Li Jin, Leming Shi, Ali J. Marian, Dao Wen Wang

**Affiliations:** ^1^Division of Cardiology, Department of Internal Medicine, Tongji Hospital, Tongji Medical College, Huazhong University of Science and Technology, Wuhan, China; ^2^Hubei Key Laboratory of Genetics and Molecular Mechanism of Cardiologic Disorders, Huazhong University of Science and Technology, Wuhan, China; ^3^Collaborative Innovation Center for Genetics and Development, School of Life Sciences, Fudan University, Shanghai, China; ^4^Center for Cardiovascular Genetics, Houston, TX, United States

**Keywords:** DCM, TTN, genetics, phenotype, prognosis

## Abstract

**Background:** Mutations in the *TTN* gene are the most common causes of dilated cardiomyopathy (DCM). The clinical significance of *TTN* gene variants remains inadequately understood.

**Methods:** Whole-exome sequencing and phenotypic characterisation were performed, and patients were followed up for a median of 44 months.

**Results:** We analyzed the association of the *TTN* variants with the clinical outcomes in a prospective study of 1,041 patients with sporadic DCM. *TTN* truncating variants (tTTN) were detected in 120 (11.5%) patients as compared with 2.4/10,000 East Asian populations in the Genome Aggregation Database (GnomAD; *p* < 0.0001). Pathogenic *TTN* missense variants were also enriched in DCM as compared with the GnomAD populations (27.6 vs. 5.9%, *p* < 0.0001). DCM patients with tTTN had a lower left ventricular ejection fraction (28.89 ± 8.72 vs. 31.81 ± 9.97, *p* = 0.002) and a lower frequency of the left bundle branch block (3.3 vs. 11.3%, *p* = 0.011) than those without or with mutations in other known causal genes (OCG). However, tTTN were not associated with the composite primary endpoint of cardiac death and heart transplantation during the follow-up period [adjusted hazard ratio (HR): 0.912; 95% confidence interval: 0.464–1.793; *p* = 0.790]. There was also no sex-dependent effect. Concomitant tTTN and pathogenic variants in OCG were present in only eight DCM patients and did not affect the outcome.

**Conclusion:** The phenotype of DCM caused by tTTN, major causes of sporadic DCM, is not distinctly different from those caused by other causal genes for DCM.

## Introduction

Dilated cardiomyopathy (DCM) is a primary disease of the myocardium characterized by an increased left ventricular end-diastolic diameter (LVEDD) and a reduced left ventricular ejection fraction (LVEF) in the absence of external causes, such as coronary artery disease (1, 2). DCM is a major cause of chronic heart failure (CHF) and the most common indication for heart transplantation (HTx) (3–5). The estimated prevalence of DCM ranges from ~1:2,700 to 1:250 individuals (6, 7). Patients with DCM exhibit a variable phenotypic expression, including age of onset, severity of the disease, and prognosis.

Primary DCM is typically a genetic disease, familial in about 1/3 of the cases and sporadic in the remainder. Mutations in over 60 genes are associated with DCM (3, 8, 9). Mutations in genes encoding sarcomere and cytoskeletal proteins are major causes of familial and sporadic DCM, although the causality of mutations is best established in the familial cases (3).

The *TTN* gene is composed of 363 exons (ENST00000589042), multiple mRNA isoforms, and codes for the largest known protein with 34,350 amino acids. *TTN* protein spans half of the sarcomere length from the Z disk to M line. Titin is implicated in conferring elasticity to sarcomere, sarcomere assembly, and mechanosensing (10–12).

Because of its enormous size, variants in the *TTN* gene, including truncating variants (tTTN), are relatively common in the general population. tTTN and missense variants are present in ~2 and 5.7% of the general population (without overt DCM), respectively (10, 13–15). tTTN are the most common causes of DCM, occurring in 10–20% of cases (11, 16). The role of *TTN* missense variants in DCM is less clear but is implicated as a modifier of the phenotype (17). Given the presence of the *TTN* variants in the general population, large datasets are necessary to determine the associations of *TTN* variants with the clinical outcomes. The results of previous genotype–phenotype correlation studies are compounded by the small sample size, cross-sectional design, and differences in the characteristics of the study populations (11, 16–20). To determine the clinical significance of *TTN* variants, we performed a prospective study of 1,041 patients with DCM who underwent phenotypic characterization and whole-exome sequencing. We determined the frequency of tTTN and missense variants in DCM cases and investigated the association of the genotypes, including topography of the variants with the clinical outcomes during a median follow-up period of 44 months.

## Materials and Methods

### Study Population

The study population was composed of 1,041 patients with a clinical diagnosis of DCM, who were recruited from Tongji Hospital Affiliated Tongji Medical College of Huazhong University of Science and Technology (Wuhan). All participants had Han ethnicity. DCM was diagnosed based on the European Society of Cardiology criteria, which included a LVEF of <50% and a LVEDD of >117% of the predicted value corrected for age and body surface area in the absence of significant coronary artery disease, primary valvular disease, or myocarditis (21).

All participants signed the informed consent prior to inclusion. The study was approved by the ethics committee of Tongji Hospital Affiliated Tongji Medical College of Huazhong University of Science and Technology and conducted in accordance with the Declaration of Helsinki and the International Conference on Harmonization Guidelines for Good Clinical Practice.

### Clinical Data and Follow-Up

Baseline demographic and clinical data were collected by interviewing patients and reviewing the clinical records of all participants. Comprehensive clinical evaluation included electrocardiography, echocardiography, coronary artery angiography, and blood biochemistry. Cardiac arrhythmias and conduction defects, namely, left bundle branch block (LBBB), atrial fibrillation (AF), and ventricular tachycardia (VT), were also recorded. All patients were followed up annually through telephone interviews and a home visit, whenever telephone follow-up was not feasible.

The primary endpoint was a composite of death due to cardiac causes and HTx. Death due to cardiac causes was defined as death as a result of cardiac pump failure, an ischemic event, or sudden cardiac death, the latter defined as death occurring in the absence of precipitating cardiovascular symptoms, as judged by clinical physicians. The secondary endpoints were all-cause mortality and recurrence of CHF. All-cause mortality was considered as death from any cause, whereas recurrence of CHF was defined as new or worsening symptoms of CHF, such as dyspnea on exertion, orthopnea, and edema.

### High-Throughput Sequencing

Genomic DNA (gDNA) was extracted from peripheral blood lymphocytes (Qiagen, Germany) and sequenced on an Illumina sequencing platform (**Supplementary Method**).

### Bioinformatics Processing

Principle Component Analysis (PCA) was performed using PLINK to detect ancestry and relatedness and exclude related subjects (22). Variants were annotated using ANNOVAR (version 2018, April 16). The population allele frequency of each variant was based on data from the Genome Aggregation Database (GnomAD). *TTN* variants were annotated to the titin meta-transcript (ENST000005859042) using LRG_391_t1. Only the variants in constitutively expressed cardiac exons [percentage spliced in (PSI) >90%] were analyzed for pathogenicity (11). Rare variants were defined as variants with a minor allele frequency (MAF) <0.1% in the GnomAD. Pathogenic variants were defined as (1) rare truncating variants (stop-gain, essential splice site, and frameshift indel) and (2) rare missense variants with a Combined Annotation-Dependent Depletion (CADD) score ≥20 (23). Other variants tagged as pathogenic in the ClinVar database were also included. All identified variants were validated through Sanger sequencing or Integrative Genomics Viewer (IGV).

### Statistical Analysis

The characteristics of the study patients were presented as mean ± SD for normally distributed continuous data; otherwise, as median [interquartile range (IQR)] for categorical data. For skewed data, numbers (percentages) were provided. Continuous variables were compared using Student's *t*-test between the two groups and the Kruskal–Wallis test, the latter when the data did not conform to normal distribution. Survival rates between the groups were compared by the Kaplan–Meier method using Cox proportional hazards regression. The association between tTTN with primary and secondary endpoints was evaluated by univariate and multivariate analyses. All statistical analyses were performed in R statistical package, version 3.5.0. A *p* < 0.05 was considered to be significant. All comparisons were two-sided.

## Results

### Clinical Characteristics

Baseline characteristics of the study population are shown in Table 1. The mean age at diagnosis and onset in patients with DCM were 55.32 ± 13.90 and 52.29 ± 13.65 years old, respectively. Upon enrollment, 69.4% of the patients had symptoms consistent with New York Heart Association (NYHA) functional classes III or IV. The mean LVEF and LVEDD, the latter indexed to body surface area (BSA), were 31.48 ± 9.87% and 38.10 ± 6.30 mm/m^2^, respectively. Baseline characteristics of the DCM population stratified by patient's sex are also shown in Table 1. The majority of DCM patients were male (*N* = 765, 73.5%). The age at onset of clinical symptoms in male patients was younger than that in female patients (51.47 ± 13.65 vs. 54.59 ± 13.41, *p* = 0.001). Moreover, female patients had a larger LVEDD indexed to BSA than male patients (41.63 ± 5.95 vs. 36.90 ± 5.97, *p* < 0.001). Except for a higher percentage of the male patients being treated with digoxin (*p* = 0.008) and angiotensin-converting enzyme inhibitor (*p* = 0.018) than female patients, pharmacological, and device-based therapy for DCM were similar between sexes.

**Table 1:** Baseline characteristics of DCM patients.

	**Overall**	**Female**	**Male**	***p***
	**(*n* = 1,041)**	**(*n* = 276)**	**(*n* = 765)**	
Age at onset (years)	52.29 ± 13.65	54.59 ± 13.41	51.47 ± 13.65	0.001
NYHA functional class III/IV, *n* (%)	722 (69.4%)	181 (65.6%)	541 (70.7%)	0.131
**Medical history**				
Hypertension, *n* (%)	536 (51.5%)	136 (49.3%)	400 (52.3%)	0.431
Diabetes, *n* (%)	176 (16.9%)	44 (15.9%)	132 (17.3%)	0.685
Stroke, *n* (%)	46 (4.4%)	13 (4.7%)	33 (4.3%)	0.92
Dyslipidemia *n* (%)	100 (9.6%)	26 (9.4%)	74 (9.7%)	0.998
**Conduction defect/Arrhythmia**				
Left bundle branch block, *n* (%)	108 (10.4%)	43 (15.6%)	65 (8.5%)	0.001
Atrial fibrillation, *n* (%)	233 (22.4%)	64 (23.2%)	169 (22.1%)	0.771
Non-sustained ventricular tachycardia, *n* (%)	129 (12.4%)	32 (11.6%)	97 (12.7%)	0.717
Sustained ventricular tachycardia, *n* (%)	10 (1.0%)	5 (1.8%)	5 (0.7%)	0.183
**Echocardiographic phenotype**				
LVEDD (mm)	6.66 ± 0.82	6.40 ± 0.75	6.76 ± 0.83	<0.001
LVEDDi (mm/m^2^)	38.10 ± 6.30	41.63 ± 5.95	36.90 ± 5.97	<0.001
LVEF (%)	31.48 ± 9.87	31.55 ± 9.55	31.45 ± 9.99	0.89
LVM (g)	296.96 ± 85.19	264.51 ± 71.24	308.68 ± 86.80	<0.001
LVMi (g/m^2^)	166.93 ± 45.82	187.84 ± 77.55	155.61 ± 44.65	<0.001
E/A ratio	1.73 ± 1.65	1.65 ± 1.59	1.75 ± 1.67	0.49
E/e' ratio	23.21 ± 14.33	25.25 ± 18.88	22.27 ± 11.58	0.05
LAD (mm)	4.56 ± 0.77	4.34 ± 0.73	4.64 ± 0.77	<0.001
**Pharmacological and device-based therapy**				
Digoxin, *n* (%)	507 (48.7%)	115 (41.7%)	392 (51.2%)	0.008
Diuretics, *n* (%)	853 (81.9%)	218 (79.0%)	635 (83.0%)	0.162
ACEI or ARB, *n* (%)	781 (75.0%)	192 (69.6%)	589 (77.0%)	0.018
Beta-blocker, *n* (%)	531 (51.0%)	132 (47.8%)	399 (52.2%)	0.245
Spironolactone, *n* (%)	799 (76.8%)	210 (76.1%)	589 (77.0%)	0.824
History of pacemaker implantation, *n* (%)	52 (5.0%)	13 (4.7%)	39 (5.1%)	0.926
History of ICD, *n* (%)	18 (1.7%)	2 (0.7%)	16 (2.1%)	0.221

*NYHA, New York Heart Association; LVEDD, left ventricular end-diastolic dimension; LVEDDi, left ventricular end-diastolic dimension index; LVEF, left ventricular ejection fraction; LVM, left ventricular mass; LVMi, left ventricular mass index; E/A ratio, early to late peak diastolic mitral flow velocity ratio; E/e' ratio, ratio of early peak diastolic mitral velocity/peak early diastolic mitral annular velocity; LAD, left atrial dimension; ACEI, angiotensin converting enzyme inhibitor; ARB, angiotensin receptor blockers; ICD, Implantable cardioverter defibrillator*.

### Burden of Variants

**Figure 1:**
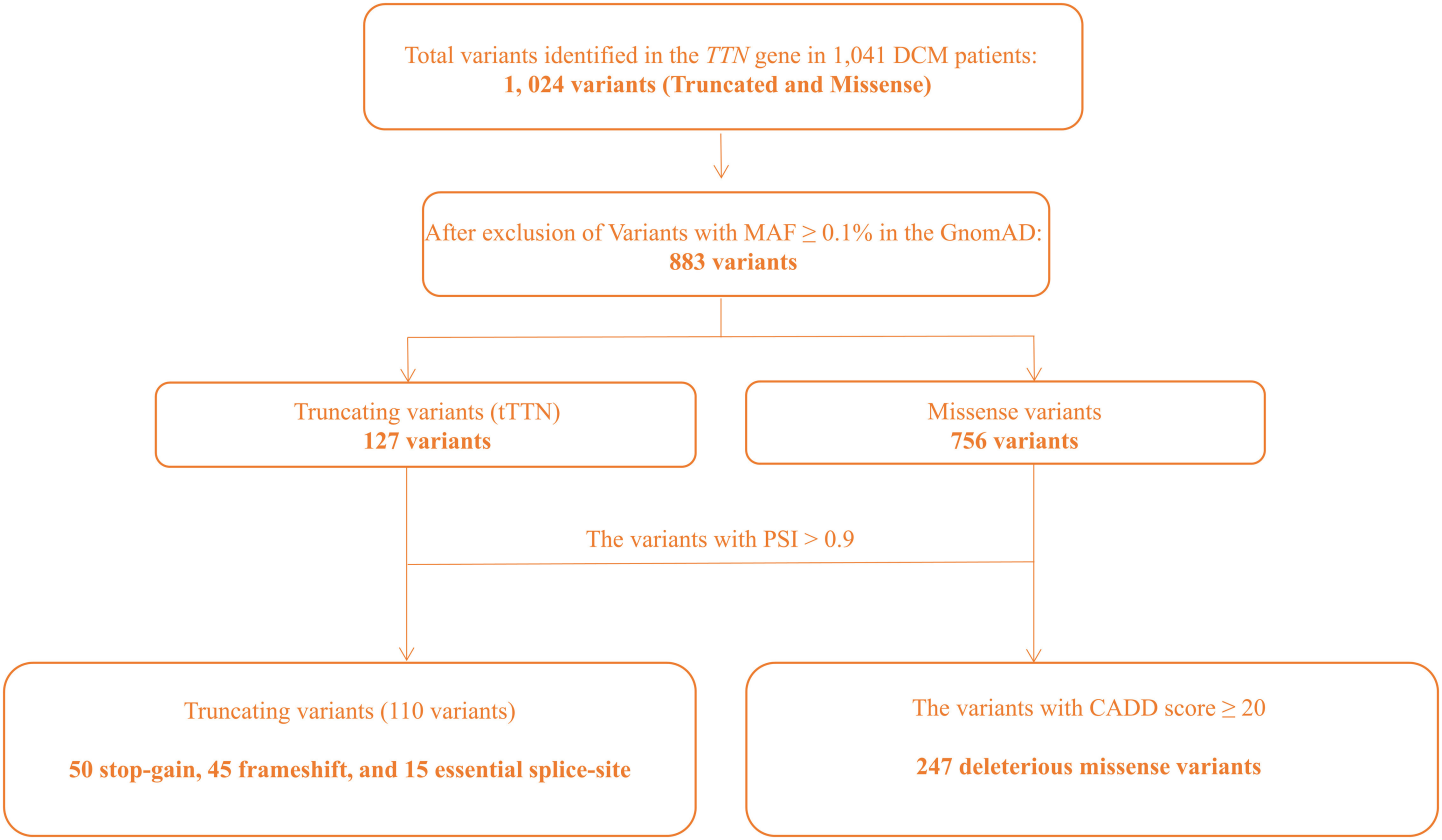
Flowchart depicting approach to identification of the pathogenic variants in the *TTN* gene.

WES was performed in 1,041 DCM patients, all of which were of Han ethnicity. No significant population stratification effect was detected per PCA (Supplementary Figure 1). Likewise, genetic relatedness analysis did not detect related subjects in the study cohort, likely reflective of the approach in selecting sporadic cases based on family history analysis (Supplementary Figure 2). The average sequence read number was 117-fold, and 49.5% of bases were covered at 100× (Supplementary Table 1). Extensive quality control was performed as outlined in Supplementary Figure 3. tTTN and *TTN* missense variants that passed the quality control assessment were further analyzed for being pathogenic, based on MAF in the GnomAD of <0.1%, PSI, and CADD scores, as delineated in Figure 1.

### *TTN* Truncating Variants

Only rare tTTN, defined as tTTN with a MAF of <0.1% in the ethnically matched GnomAD population, were considered potentially pathogenic. One hundred twenty-seven tTTN had a MAF of <0.1% of which 110 (86.6%) had a PSI score of ≥0.9, which were considered pathogenic variants according to the 2015 ACMG guidelines. The pathogenic tTTN variants comprised 50 (45.5%) stop-gain, 45 (40.9%) frameshift, and 15 (13.6%) essential splice site variants. The majority of the pathogenic tTTN were novel (81/110, 73.6%), which are listed in Supplementary Table 2, and all were heterozygous. Pathogenic variants were detected in 11.5% (120/1,041) of the DCM patients. Compared with the ethnically matched GnomAD population (2.4/10,000), tTTN were enriched in the DCM cohort (*p* < 0.0001). Seven tTTN were identified in more than one DCM patient. These variants were enriched in the DCM patients as compared with the East Asian population in the GnomAD (Supplementary Table 3).

### *TTN* Missense Variants

To ascertain the pathogenicity of the missense variants identified in the *TTN* gene, the ClinVar database was used to identify the deleterious variants that were recorded as “pathogenic” or “likely pathogenic.” In addition, rare missense variants that had a CADD score of ≥20 were defined as potential pathogenic variants. A total of 247 variants were identified in 27.6% cases (287/1,041), which suggested enrichment of the pathogenic missense variants (PMVs) in the sporadic DCM population, compared with the ethnically matched GnomAD population (5.9 vs. 27.6%, *p* < 0.0001). The clinical characteristic of the patients at the baseline stratified by PMV status was shown in Supplementary Table 4. The mean age at onset of symptoms in patients with PMVs was older than that in patients without. Apart from the higher use of diuretics, there were no significant differences in medication use between those with and without PMVs.

### *tTTN* and the Clinical Phenotype at the Baseline

Table 2 shows a comparison of the clinical data between DCM patients who carried tTTN (*n* = 120) vs. those who did not (*n* = 921). DCM patients with the tTTN had a lower LVEF (28.89 ± 8.72) than those without (31.81 ± 9.97, *p* = 0.002). In addition, the prevalence of conduction defects, namely, LBBB, was lower in those with tTTN variants than in those without (4/120 vs. 104/921, *p* = 0.011). There was no association with a specific type of tTTN, namely, non-sense, frameshift, and essential splice site variants, and the presence of LBBB, AF, or VT between the three groups (Supplementary Table 5). Furthermore, there were no significant differences in the use of cardiovascular drugs or cardioverter defibrillator between DCM patients with and without tTTN, except for the implantation of pacemaker, which was more common in those without tTTN (Table 2). The clinical data of DCM patients with tTTN stratified by patient's sex are shown in Supplementary Table 6. Apart from the echocardiographic phenotypes of LVM index (LVMi), and LVEDD index (LVEDDi), which were different between male and female patients, there were no significant differences between male and female patients in the clinical DCM phenotypes of patients with tTTN.

**Table 2:** Clinical feature of DCM stratified by tTTN status.

	**tTTN-absent**	**tTTN-present**	***P***
	**(*n* = 921)**	**(*n* = 120)**	
Male, *n* (%)	667 (72.4%)	98 (81.7%)	0.041
Age at onset (years)	52.53 ± 13.75	50.45 ± 12.75	0.116
NYHA functional class III/IV, *n* (%)	628 (68.2%)	94 (78.3%)	0.031
**Medical history**				
Hypertension, *n* (%)	504 (54.7%)	32 (26.7%)	<0.001
Diabetes, *n* (%)	161 (17.5%)	15 (12.5%)	0.215
Stroke, *n* (%)	45 (4.9%)	1 (0.8%)	0.072
Dyslipidemia *n* (%)	90 (9.8%)	10 (8.3%)	0.735
**Conduction defect/Arrhythmia**				
Left bundle branch block, *n* (%)	104 (11.3%)	4 (3.3%)	0.011
Atrial fibrillation, *n* (%)	202 (21.9%)	31 (25.8%)	0.397
Non-sustained ventricular tachycardia, *n* (%)	111 (12.1%)	18 (15.0%)	0.439
Sustained ventricular tachycardia, *n* (%)	7 (0.8%)	3 (2.5%)	0.180
**Echocardiographic phenotype**				
LVEDD (mm)	6.67 ± 0.84	6.59 ± 0.68	0.285
LVEDDi (mm/m^2^)	38.20 ± 6.40	37.43 ± 5.64	0.484
LVEF (%)	31.81 ± 9.97	28.89 ± 8.72	0.002
LVM (g)	299.64 ± 87.32	276.51 ± 63.43	0.006
LVMi (g/m^2^)	165.49 ± 56.85	151.57 ± 51.88	0.157
E/A ratio	1.74 ± 1.73	1.57 ± 0.84	0.408
E/e' ratio	23.44 ± 14.66	21.07 ± 10.81	0.315
LAD (mm)	4.56 ± 0.76	4.58 ± 0.85	0.791
**Pharmacological and device-based therapy**				
Digoxin, *n* (%)	445 (48.3%)	62 (51.7%)	0.553
Diuretics, *n* (%)	752 (81.7%)	101 (84.2%)	0.584
ACEI or ARB, *n* (%)	693 (75.2%)	88 (73.3%)	0.732
Beta-blocker, *n* (%)	471 (51.1%)	60 (50.0%)	0.890
Spironolactone, *n* (%)	706 (76.7%)	93 (77.5%)	0.927
History of pacemaker implantation, *n* (%)	51 (5.5%)	1 (0.8%)	0.045
History of ICD, *n* (%)	16 (1.7%)	2 (1.7%)	0.975

*NYHA, New York Heart Association; LVEDD, left ventricular end-diastolic dimension; LVEDDi, left ventricular end-diastolic dimension index; LVEF, left ventricular ejection fraction; LVM, left ventricular mass; LVMi, left ventricular mass index; E/A ratio, early to late peak diastolic mitral flow velocity ratio; E/e' ratio, ratio of early peak diastolic mitral velocity/peak early diastolic mitral annular velocity; LAD, left atrial dimension; ACEI, angiotensin converting enzyme inhibitor; ARB, angiotensin receptor blockers; ICD, Implantable cardioverter defibrillator*.

The distribution of tTTN across the *TTN* protein was non-random, as the vast majority of the tTTN were located in the A-band (85/110, 77.5%) (Supplementary Figure 4). Because tTTN located at the A-band have been associated with severity of DCM (16), clinical findings at the baseline are compared between DCM patients who carried tTTN in the A-band and outside. The mean age of onset of DCM in patients with tTTN located in the A-band was 50.04 ± 13.19 years, which was not significantly different from the mean age of patients carrying tTTN located outside of the A-band (51.85 ± 11.21, *p* = 0.52). However, patients with tTTN in the A-band had a larger LVEDDi than patients with tTTN outside of the A-band (38.59 ± 5.80 vs. 33.72 ± 3.03, *p* = 0.022). In terms of pharmacological and device-based therapy, there was no significant difference between patients with different location tTTN.

### *tTTN* and Clinical Outcomes

DCM patients were followed up for a median duration of 44 months (IQR: 30–61 months). Twenty-six patients (2.6%) received HTx during the follow-up period. A total of 406 patients (39.0%) met the primary composite endpoint of cardiovascular mortality and HTx. The primary composite outcome occurred in 51/120 (42.5%) DCM patients with tTTN and 355/921 (38.5%) patients without tTTN (*p* = 0.403).

**Figure 2:**
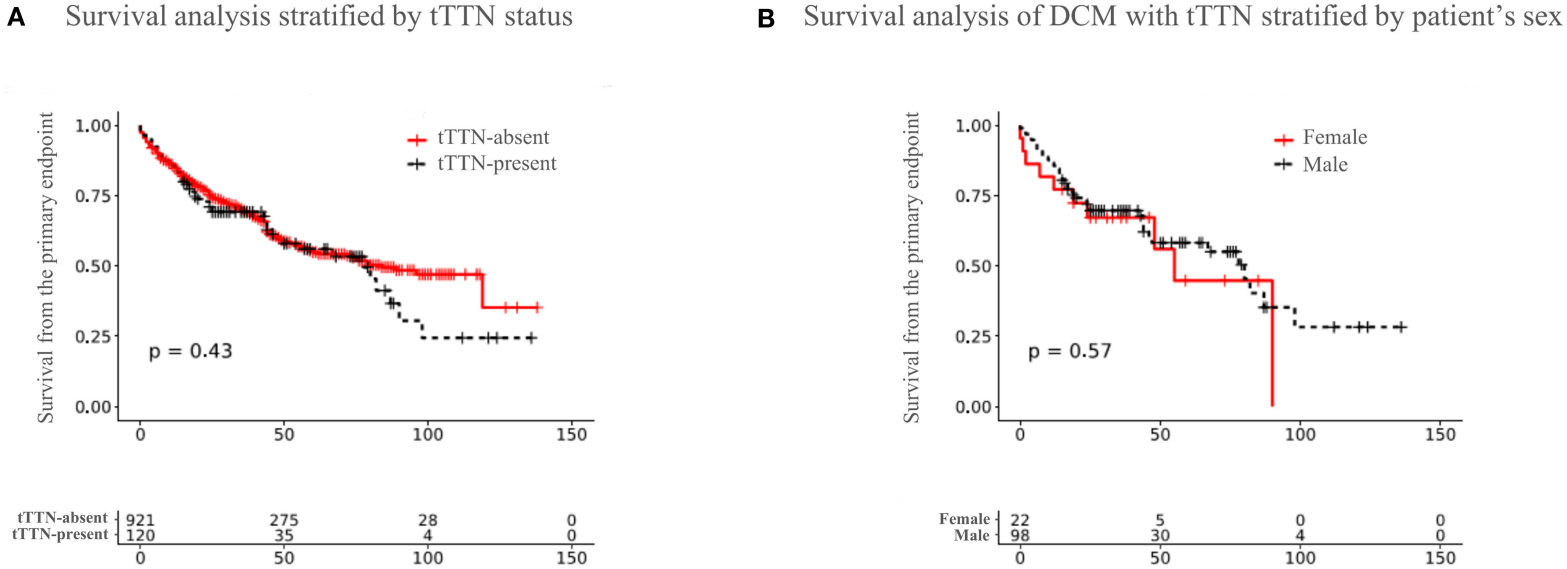
Outcome of DCM patients in our cohort. **(A)** Survival curves showing freedom from the primary endpoint in the tTTN-present DCM and tTTN-absent DCM patients. Red color represents the tTTN-absent, and black color represents the tTTN-present DCM patients. **(B)** Survival curves comparing freedom from the primary endpoint between male and female DCM patients carrying tTTN. Red color represents female, and black color represents male.

A univariate Cox proportional hazards regression model for the endpoint is shown in Supplementary Table 7. The variables that reached a significant threshold of *p* < 0.1 were considered for inclusion in multivariate analysis. Survival curves comparing the freedom from the primary composite endpoint for patients with and without tTTN are shown in Figure 2A. A univariate Cox proportional hazard model showed that patients with tTTN compared with those without had a hazard ratio (HR) of 1.120 (95% confidence interval: 0.838–1.510; *p* = 0.434) for the primary endpoint. Multivariate analysis did not detect an association between the tTTN status and the primary endpoint (adjusted HR: 0.912; 95% confidence interval: 0.464–1.793; *p* = 0.790). Likewise, sex-dependent analysis of survival did not show a significant difference between male and female DCM patients carrying tTTN (Figure 2B).

Regarding the second endpoint, 61 (50.8%) patients with tTTN and 444 (48.2%) without tTTN experienced the second endpoint composited of all-cause mortality and recurrence of CHF (HR: 1.120; 95% confidence interval: 0.860–1.470; *p* = 0.392). After multivariate adjustment, there were still no significant differences in the second endpoint (adjusted HR: 0.910; 95% confidence interval: 0.520–1.620; *p* = 0.755).

### Effect of *TTN* on Prognosis Independent of Other DCM Genes

The presence of pathogenic variants was also analyzed in 36 other known causal genes (OCG) for DCM (9). According to the ACMG criterion, 60 pathogenic variants (Supplementary Table 8) were identified in 79 patients. Eight patients had concomitant tTTN and a pathogenic variant in OCG. Supplementary Table 9 shows the comparison of the clinical characteristics between DCM patients carrying tTTN according to the presence or absence of a second pathogenic variant in the OCG (tTTN+/OCG+ vs. tTTN+/OCG–). The Kaplan–Meier survival analysis showed that there was no statistical difference between tTTN+/OCG+ and tTTN+/OCG– (*p* = 0.650).

To determine whether DCM associated with tTTN variants had different phenotypic characteristics than DCM associated with pathogenic variants in OCG, the clinical characteristics of DCM patients with tTTN without pathogenic variants in the OCG (TTTN+/OCG–) and patients with pathogenic variants in OCG but no tTTN were compared (Supplementary Table 10). Arrhythmia, namely, LBBB, was more common in DCM patients with pathogenic variants in the OCG only than in patients with tTTN only. In addition, DCM patients in the tTTN+/OCG– group had a lower LVEF than those in the tTTN–/OCG+ group. Moreover, the rate of pacemaker implantation was higher in the tTTN–/OCG+ group. The Kaplan–Meier survival analysis showed no difference in the survival rates between tTTN+/OCG– and tTTN–/OCG+ for the primary endpoint (*p* = 0.480).

Survival analysis was repeated after the exclusion of 79 patients who had pathogenic variants in the OCG, to assess the independent effect of tTTN on prognosis. The difference between the unadjusted and adjusted HRs for the primary endpoint in those with and without tTTN was not significant (unadjusted HR: 1.170; *p* = 0.300 and adjusted HR: 0.993; *p* = 0.985). These results further suggested that tTTN were not a significant prognostic indicator in sporadic DCM cases.

## Discussion

This is the first genetic screening of a large cohort of sporadic DCM patients in the Han Chinese population and likely the largest longitudinal dataset analyzing the clinical significance of the *TTN* variants in patients with DCM. Whole-exome sequencing of 1,041 DCM patients led to the identification of tTTN in 11.5% and PMVs in 27.6% of the DCM patients, indicating a significant enrichment as compared with the ethnically matched GnomAD population. The majority (73.6%) of the tTTN identified in this population were novel, likely reflective of the ethnic background of the study population. Overall, the phenotypic characteristics of DCM patients carrying the tTTN were not different from DCM caused by other causes, including pathogenic variants in OCG for DCM. There were some exceptions, such as LVEF, which was lower in DCM patients with tTTN than in those without. Likewise, LBBB was less common in DCM patients with tTTN than in those without tTTN. The composite primary endpoint of cardiac death and HTx was not significantly different between DCM patients with and without tTTN. Similarly, there were no significant differences in the clinical phenotype of DCM in male and female patients with tTTN. Overall, the data suggest that DCM associated with tTTN is phenotypically not that distinct from DCM caused by other genetic causes or non-genetic causes.

### Burden of *TTN* Variants in DCM Patients

The prevalence of tTTN in our sporadic DCM population (11.5%) was similar to that reported in sporadic DCM, which varies from 11 to 18% (9, 16, 24). A higher prevalence of tTTN (21.4%) has been observed in Japanese patients with sporadic DCM (25). tTTN seem to be more common in familial DCM, as they have been identified in 19–25% of familial DCM (9, 16, 24). The original report by Herman et al. noted a tTTN frequency ranging from 8 to 40% in the three clinical cohorts (16). The differences among different studies might reflect differences in the sample sizes of the studies, the ethnic backgrounds of the study populations, and the technical differences in mutation analysis.

Roberts et al. reported that almost half of the identified tTTN in their population was located in exons with a low PSI (fraction of mRNAs that represent the inclusion isoform) (11). tTTN with low PSI were referred to as alleles with a low probability of pathogenicity. In contrast, tTTN that affect all transcripts (transcripts having high PSI) were considered pathogenic and likely pathogenic. The latter group of tTTN is present at a frequency of about 0.35% in the general population [averaged over the 1000 Genomes Project, NHLBI GO Exome Sequencing Project (ESP), and ExAC] (26). In the present study, pathogenic tTTN, based on PSI >0.9, were detected in 11.5% of the sporadic DCM population.

Determining the pathogenicity of the *TTN* missense variants is more challenging, because of the abundance of such variants in the general population. The high prevalence of missense variants in *TTN* has been observed in DCM populations, and such variants are considered as potential modifiers of the clinical phenotypes (20, 27). In accord with the findings of the present study, no significant differences in the clinical phenotypes in DCM patients carrying bioinformatically “severe” *TTN* missense variants as compared with the non-carriers have been noted (27). In the present study, modest differences were noted, including the older age of onset of symptoms in DCM patients with PMVs as compared with those without. Otherwise, there were no significant differences in other clinical phenotypes between the two groups, which suggest that the effect sizes of the *TTN* missense variants are relatively modest, if any.

### *tTTN* and Sex

Although, there is a common theme that male DCM patients carrying tTTN would have worse prognosis than female DCM patients. The association between tTTN and sex becomes complicated in previous studies (11, 16, 18, 19, 28, 29). Akhtar et al. reported a large cohort of 537 DCM with tTTN, in which the male patients had worse outcomes than the female patients (29). In the present study, we have shown that there is no significant difference between male patients and female patients for clinical phenotypes and outcomes, which is consistent with the results reported by Tayal et al. (28). In our cohort, only 18.3 patients with tTTN were female, and fewer events would limit the statistical power. In addition, DCM, as an autosomal dominant disease, should have a male-to-female ratio of 1:1, which is inconsistent with the actual male-to-female ratio. The inconsistency implies that other modifiers may have effects on the development of phenotypes and outcomes in DCM patients with tTTN.

### Clinical Significance of *tTTN* in DCM Patients

The clinical impact of tTTN has remained unsettled and varies among studies. A previous study showed an association between tTTN and the clinical outcome, specifically, patients with tTTN had less severe heart failure at presentation and more were amenable to standard therapy than those with DCM associated with the *LMNA* mutations or those with idiopathic DCM (19). In addition, tTTN have been shown to be an independent determinant of left ventricular reverse remodeling (25). Others have reported no significant clinical outcome differences between DCM patients with tTTN and without tTTN (18, 28). In the present study, which is the largest thus far, we did not detect an increased risk for adverse clinical outcomes in DCM patients with tTTN as compared with those without. The presence of concomitant pathogenic variants is typically associated with a worse phenotype. Likewise, patients with *TTN* missense and *LMNA* variants have been shown to have a more severe early-onset phenotype (17). In the present study, the concomitant presence of pathogenic variants in OCG for DCM was detected in eight patients. However, the phenotype and outcome did not show a significant difference between tTTN+/OCG+ and tTTN+/OCG–. Finally, the topography of the tTTN on titin protein has been implicated in the pathogenicity and severity of the DCM phenotype (30, 31). In the present study, there were no notable differences in the clinical phenotypes in DCM patients with tTTN in the A-band and those with tTTN outside the A-band. Previous studies have reported conflicting associations between arrhythmia with tTTN (28, 32). The largest cohort of 537 DCM patients with tTTN to date reported by Akhtar et al. confirmed that tTTN are characterized by high prevalence of atrial and ventricular arrhythmia (29). In this study, we did not find an increased risk of atrial or ventricular arrhythmia in tTTN carriers compared with those without tTTN. The low arrhythmia rate in our study may account for the result; thus, a larger cohort is needed to focus on arrhythmia in DCM with tTTN.

Although this is a large cohort with 1,041 patients to assess the effect of *TTN* variants on long-term prognosis, it has several limitations. First, this is a single-center study, which would introduce inclusion bias. Second, there is a gap between the clinical management of study patients and the optimal treatment, which may have an effect on long-term prognosis. Third, the findings in this study require replication in an independent cohort, and it is necessary to perform a deeper phenotypic characterization (i.e., arrhythmia, cardiac endophenotypes).

## Data Availability Statement

The data presented in the study are deposited in the gsa-human repository, accession number https://bigd.big.ac.cn/gsa-human/browse/HRA000431.

## Ethics Statement

The studies involving human participants were reviewed and approved by the ethics committee of Tongji Hospital Affiliated Tongji Medical College of Huazhong University of Science and Technology. The patients/participants provided their written informed consent to participate in this study.

## Author Contributions

LX developed the study concept and design, interpreted the data, and drafted the manuscript. CL, YS, YC, HW, DH, TY, and XL performed the experiments and analyzed the data. LJ, LS, AM, and DW supervised the design of the study and revised the manuscript. All authors contributed to the article and approved the submitted version.

## Conflict of Interest

The authors declare that the research was conducted in the absence of any commercial or financial relationships that could be construed as a potential conflict of interest.
