# Supplementary material for: Clinical Significance of Variants in the TTN Gene in a Large Cohort of Patients With Sporadic Dilated Cardiomyopathy
Source: Front Cardiovasc Med. 2021 Apr 30;8:657689. doi: 10.3389/fcvm.2021.657689 (PMC8120103; doi:10.3389/fcvm.2021.657689)
Supplement: Supplementary file 1 [file Data_Sheet_1.docx]

**Clinical Significance of Variants in the *TTN* Gene in a Large Cohort of Patients with Sporadic Dilated Cardiomyopathy**

Lei Xiao^1,2^, MD; Chenze Li^1,2^, MD; Yang Sun^1,2^, MD; Yanghui Chen^1,2^, MD; Haoran Wei^1,2^; MD; Dong Hu^1,2^, MD; Ting Yu^1,2^, BN; Xianqing Li^1,2^, MD; Li Jin^3^, PhD; Leming Shi^3^, PhD; Ali J. Marian^4^, MD, and Dao Wen Wang^1, 2, 3^, MD, PhD

Supplementary Methods.

Figure S1. Ancestry analysis.

Figure S2. Relatedness analysis.

Figure S3. The workflow of bioinformatic filtering for the qualified variants.

Figure S4. Location of the tTTN found in our study.

Table S1. Summary of the sequence mapping coverage.

Table S2. The pathogenic tTTN identified in the cohort of DCM patients.

Table S3. Recurring tTTN in the cohort.

Table S4. Clinical Feature of DCM stratified by pathogenic missense variants (PMV) status.

Table S5. Cardiac arrhythmia and conduction defects in tTTN stratified by variant type.

Table S6. Clinical Feature of DCM patients with tTTN Stratified by sex.

Table S7. Results of univariable Cox Proportional hazard modelling predicting end-point.

Table S8. The list of pathogenic variants in the other causal genes of DCM.

Table S9. Table S9. Phenotype in those with tTTN vs those with tTTN and pathogenic variants in OCG.

Table S10. Clinical Feature of DCM Stratified by pathogenic variants status.

**Supplementary Methods:**

*Whole Exome Sequence (WES) on* *Illumina HiSeq X and NovaSeq System*

All gDNA samples were of high quality with an optical density (OD) 260/280 ratio ranging from 1.8 to 2.0. In total, 1 μg of gDNA was sheared into 250-bp to 300-bp fragments using the Covaris S220 sonicator (Covaris), sheared DNA were checked using Agarose Gel Electrophoresis and purified using AMPure XP Beads (Beckman). Then End-Repair, 3’ End A-tailing, Adaptor-Ligation, and Amplification were performed, AMPure XP Beads were used to purify DNA after each enzymatic reaction. After final purification, size distribution and quantification of amplified library were determined using Agilent 2100 Bioanalyzer (Agilent) and Qubit (Invitrogen). Then, 750 ng of the Illumina paired-end pre-capture library were hybridized to Agilent SureSelectXT Human All Exome V6 capture probes. After hybridization, target regions were purified using streptavidin-coated magnetic beads and each library was amplified to add index tags. Captured libraries were quantified using the Agilent 2200 Bioanalyzer and pooled such that each index-tagged sample was present in equimolar amounts in the final sequencing sample pool (at concentration of 2 nM). The pooled samples were subjected to sequencing using an Illumina X10 or NovaSeq sequencer (Illumina) in a 2 × 150 paired-end format.

*Data processing and quality control*

The WES data obtained from Illumina HiSeq X and NovaSeq System were processed according to the Genome Analysis Toolkit (GATK) Best Practices recommendations.(1) In brief, the sequence reads of each individual were aligned to the hg19 human reference genome using the Burrows–Wheeler Aligner.(2) Picard was used to sort BAM files and mark duplicates, which are read pairs originating from the same original DNA fragments through some artifactual processes such as PCR. insert/deletions (indels) realignment, base quality recalibration, variant calling, and variant quality score recalibration was performed using GATK in both cases and controls. Tagged variants identified other than ‘‘PASS’’ were not considered in the downstream analysis. Variant Call Format manipulation was done using VCFtools. Genotypes were considered as missing if the depth of coverage (DP) less than 10. We then screened out multiallelic variants or variants missing in more than 20% of all samples from downstream analysis, considering the ambiguity in calling quality. Furthermore, we filtered for variants with an average allele balance ≧ 0.2 for heterozygous genotypes.


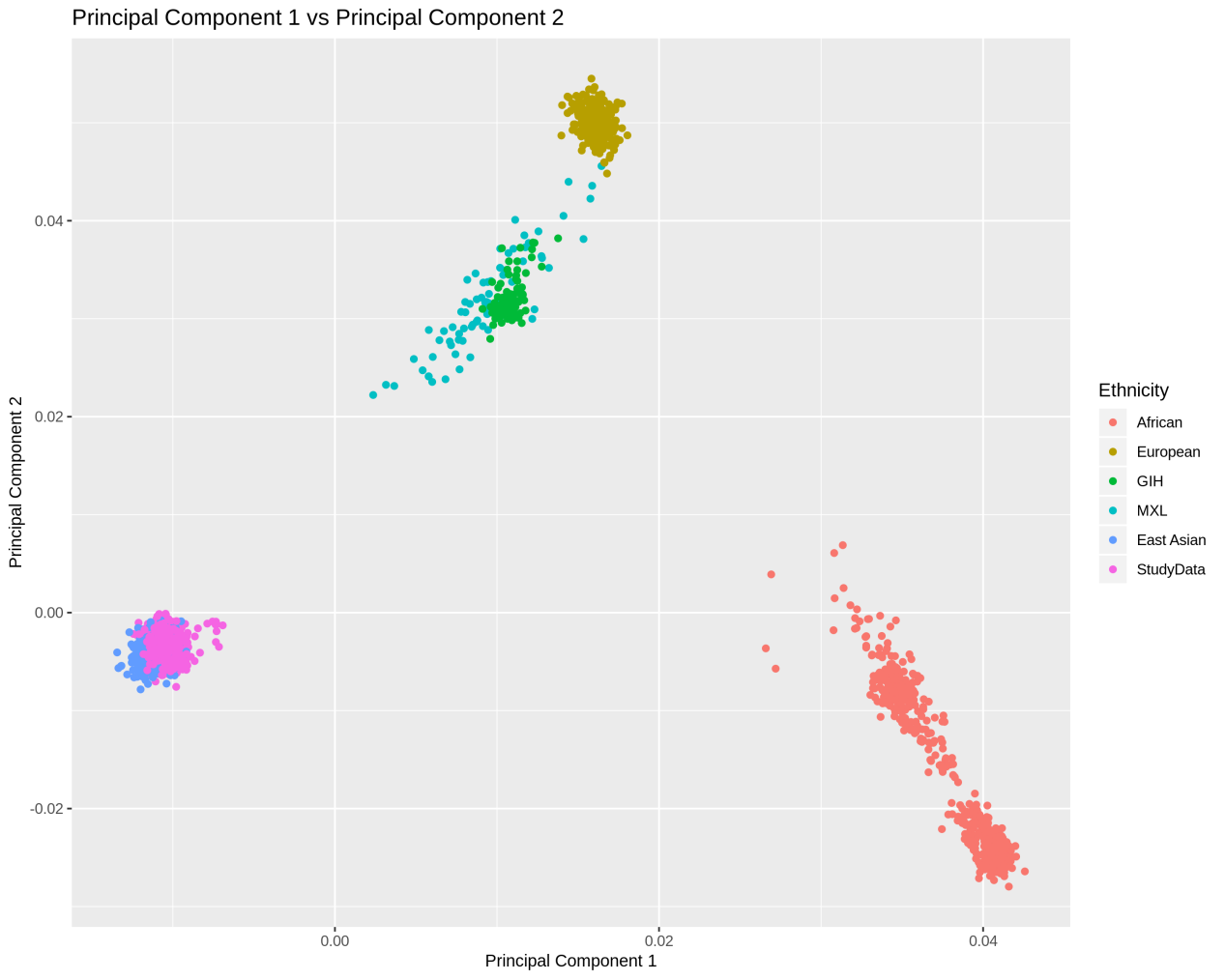


**Figure S1.** Ancestry analysis of the study subjects showing the study subjects were of east Asian ancestry and no significant population stratification.


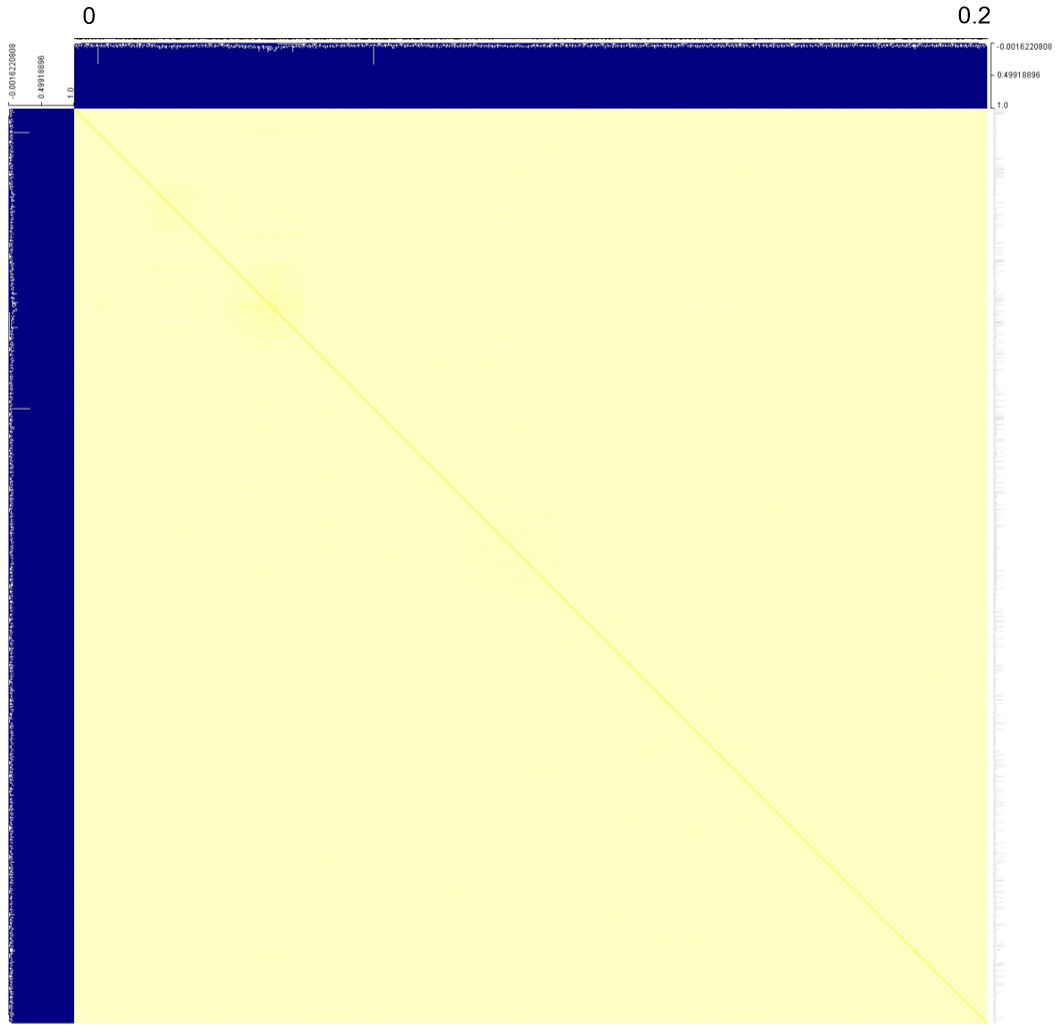


**Figure S2.** Relatedness analysis of study participants showing there were no related subjects in study group.


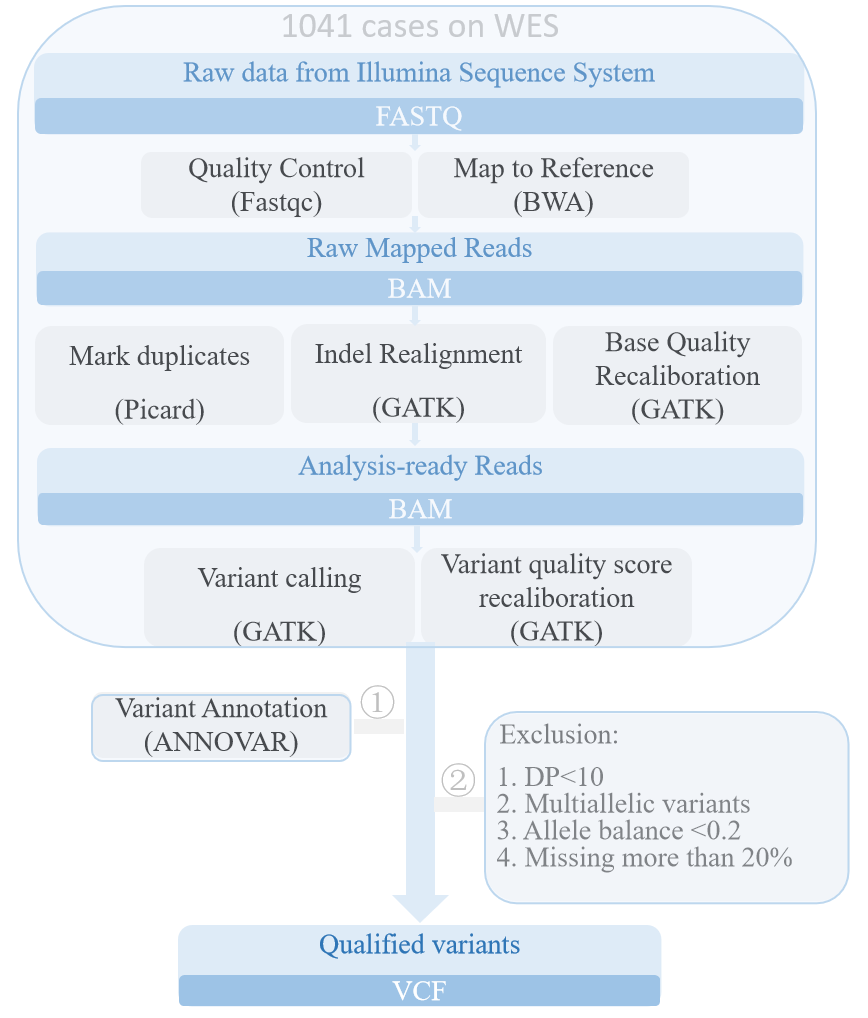


**Figure S3.** The workflow of bioinformatic filtering for the qualified variants.


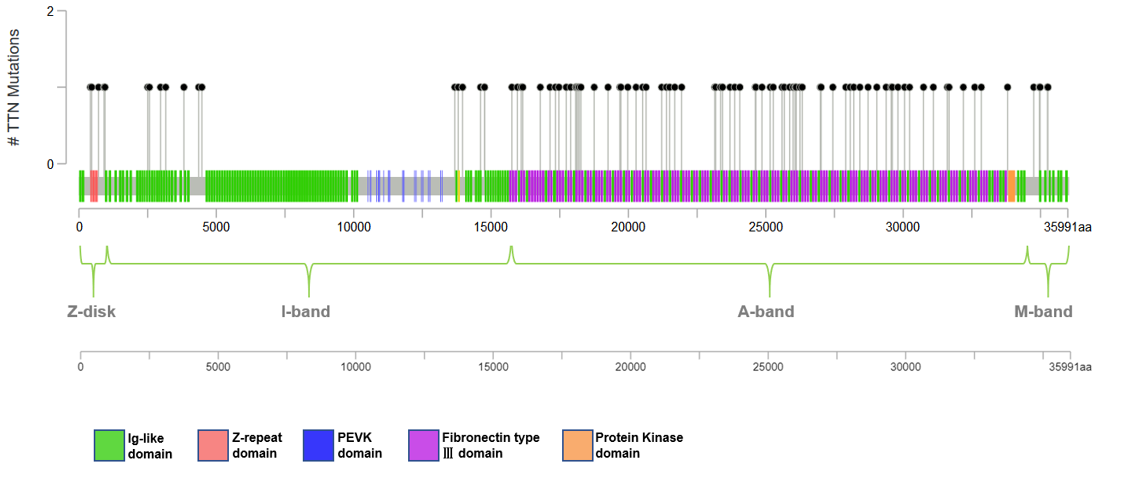


**Figure S4.** **Location of the TTNtv in the study population.**

The non-random distribution of TTNtv on protein level. Different colors represent different domain. Red is Ig-like domain, Brown is Z-repeat domain, Blue is PEVK domain, Orange is Protein kinase domain, Purple is Fibronectin type Ⅲ domain.

| **Table S1. Summary of the sequence read coverage.** | |
| --- | --- |
|  | **Coverage** |
| mean coverage X | 117X |
| coverage_1X_% | 98.90% |
| coverage_20X_% | 97.10% |
| coverage_50X_% | 87.60% |
| coverage_100X_% | 49.45% |

| **Table S2. The pathogenic *tTTN* identified in the cohort of DCM patients.** | | | | | | | |
| --- | --- | --- | --- | --- | --- | --- | --- |
| **No.** | **Coding change** | **Protein change** | **PSI** | **Novelty** | **GnomAD** | **Confirmed** | **ACMG** |
| 2 | c.105800_105801del | p. T35267fs | 1 | novel | NA | IGV | PVS1+PM2+PM6+PP4 |
| 1 | c.C105754T | p. R35252X | 1 | rs886043924 | 8.14E-06 | Sanger | PVS1+PM2+PP3+PP4+PP5 |
| 1 | c.C104947T | p. Q34983X | 1 | rs991187915 | NA | Sanger | PVS1+PM2+PP3+PP4 |
| 1 | c.104874delT | p. N34958fs | 1 | novel | NA | Sanger | PVS1+PM2+PM6+PP4 |
| 1 | c.C104254T | p. Q34752X | 1 | novel | NA | Sanger | PVS1+PM2+PM6+PP3+PP4 |
| 1 | c.101398delA | p. R33800fs | 1 | novel | NA | Sanger | PVS1+PM2+PM6+PP4 |
| 1 | c.G98529A | p. W32843X | 1 | novel | NA | Sanger | PVS1+PM2+PM6+PP3+PP4 |
| 1 | c.98495dupT | p. I32832fs | 1 | novel | NA | IGV | PVS1+PM2+PM6+PP4 |
| 1 | c.98416dupG | p. D32806fs | 1 | novel | NA | Sanger | PVS1+PM2+PM6+PP4 |
| 1 | c.C97813T | p. Q32605X | 1 | — | 4.1E-06 | Sanger | PVS1+PM2+PP3+PP4 |
| 1 | c.96569delC | p. P32190fs | 1 | novel | NA | IGV | PVS1+PM2+PM6+PP4 |
| 1 | c.C95008T | p. R31670X | 1 | rs1322596650 | 4.07E-06 | IGV | PVS1+PM2+PP3+PP4+PP5 |
| 1 | c.C94816T | p. R31606X | 1 | rs1060500435 | NA | Sanger | PVS1+PM2+PP4+PP5 |
| 1 | c.93291_93300del | p. G31097fs | 1 | novel | NA | IGV | PVS1+PM2+PM6+PP4 |
| 1 | c.G92219A | p. W30740X | 1 | novel | NA | Sanger | PVS1+PM2+PM6+PP3+PP4 |
| 1 | c.92057dupA | p. N30686fs | 1 | novel | NA | Sanger | PVS1+PM2+PM6+PP4 |
| 1 | c.C90697T | p. R30233X | 1 | rs1553539391 | NA | Sanger | PVS1+PM2+PP3+PP4+PP5 |
| 1 | c.90244dupA | p. I30082fs | 1 | novel | NA | IGV | PVS1+PM2+PM6+PP4 |
| 1 | c.G90153A | p. W30051X | 1 | novel | NA | Sanger | PVS1+PM2+PM6+PP3+PP4 |
| 1 | c.89425delC | p. R29809fs | 1 | novel | NA | Sanger | PVS1+PM2+PM6+PP4 |
| 1 | c.88987dupA | p. S29663fs | 1 | novel | NA | IGV | PVS1+PM2+PM6+PP4 |
| 1 | c.88812_88813del | p. Y29604fs | 1 | novel | NA | Sanger | PVS1+PM2+PM6+PP4 |
| 1 | c.88688delG | p. G29563fs | 1 | novel | NA | Sanger | PVS1+PM2+PM6+PP4 |
| 1 | c.88137delT | p. D29379fs | 1 | novel | NA | IGV | PVS1+PM2+PM6+PP4 |
| 1 | c.87706+1->A |  | 1 | novel | NA | Sanger | PVS1+PM2+PM6+PP4 |
| 2 | c.87119-1G>T |  | 1 | novel | NA | Sanger | PVS1+PM2+PM6+PP4 |
| 2 | c.86162_86163del | p. Y28721fs | 1 | novel | NA | Sanger | PVS1+PM2+PM6+PP4 |
| 1 | c.86127dupA | p. Y28710fs | 1 | novel | NA | Sanger | PVS1+PM2+PM6+PP4 |
| 1 | c.C85267T | p. R28423X | 1 | rs769664554 | 8.15E-06 | IGV | PVS1+PM2+PP3+PP4 |
| 1 | c.G84602A | p. W28201X | 1 | novel | NA | Sanger | PVS1+PM2+PM6+PP3+PP4 |
| 1 | c.84215delT | p. I28072fs | 1 | novel | NA | Sanger | PVS1+PM2+PM6+PP4 |
| 1 | c.83838dupT | p. P27947fs | 1 | novel | NA | Sanger | PVS1+PM2+PM6+PP4 |
| 1 | c.83730delG | p. W27910X | 1 | novel | NA | Sanger | PVS1+PM2+PM6+PP4 |
| 1 | c.82319_82329del | p. Y27440fs | 1 | novel | NA | Sanger | PVS1+PM2+PM6+PP4 |
| 4 | c.C81037T | p. R27013X | 1 | rs869038795 | NA | IGV | PVS1+PM2+PP3+PP4+PP5 |
| 1 | c.80955delT | p. V26985fs | 1 | Clinvar | NA | Sanger | PVS1+PM2+PM6+PP4 |
| 1 | c.C78979T | p. R26327X | 1 | ra1419374180 | NA | Sanger | PVS1+PM2+PP4+PP5 |
| 1 | c.78728_78731del | p. K26243fs | 1 | novel | NA | Sanger | PVS1+PM2+PM6+PP4 |
| 1 | c.78316delA | p. T26106fs | 1 | novel | NA | Sanger | PVS1+PM2+PM6+PP4 |
| 1 | c.77978delC | p. P25993fs | 1 | novel | NA | IGV | PVS1+PM2+PM6+PP3+PP4 |
| 1 | c.C77599T | p. Q25867X | 1 | novel | NA | Sanger | PVS1+PM2+PM6+PP3+PP4 |
| 1 | c.A77020T | p. R25674X | 1 | novel | NA | Sanger | PVS1+PM2+PM6+PP4 |
| 1 | c.76768_76769del | p. K25590fs | 1 | novel | NA | Sanger | PVS1+PM2+PM6+PP4 |
| 1 | c.75813delA | p. E25271fs | 1 | novel | NA | IGV | PVS1+PM2+PM6+PP4 |
| 1 | c.C75469T | p. R25157X | 1 | rs1553603394 | NA | Sanger | PVS1+PM2+PP3+PP4+PP5 |
| 1 | c.C74579A | p. S24860X | 1 | novel | NA | IGV | PVS1+PM2+PM6+PP3+PP4 |
| 1 | c.G73910A | p. W24637X | 1 | novel | NA | Sanger | PVS1+PM2+PM6+PP3+PP4+PP5 |
| 1 | c.C73846T | p. R24616X | 1 | rs794729284 | 4.08E-06 | IGV | PVS1+PM2+PP3+PP4+PP5 |
| 1 | c.73409_73410insCTTT | p. L24470fs | 1 | novel | NA | Sanger | PVS1+PM2+PM6+PP4 |
| 1 | c.C72161G | p. S24054X | 1 | novel | NA | IGV | PVS1+PM2+PM6+PP4 |
| 1 | c.C71602T | p. R23868X | 1 | rs397517689 | 4.08E-06 | IGV | PVS1+PM2+PP3+PP4+PP5 |
| 1 | c.71084_71085del | p. E23695fs | 1 | novel | NA | IGV | PVS1+PM2+PM6+PP4 |
| 1 | c.70281delT | p. F23427fs | 1 | novel | NA | Sanger | PVS1+PM2+PM6+PP4 |
| 1 | c.70215dupT | p. N23406_R23407delinsX | 1 | rs1553614378 | NA | IGV | PVS1+PM2+PP4+PP5 |
| 1 | c.C70051T | p. R23351X | 1 | rs1060500575 | NA | IGV | PVS1+PM2+PP4+PP5 |
| 1 | c.T69522A | p. Y23174X | 1 | novel | NA | Sanger | PVS1+PM2+PM6+PP4 |
| 1 | c.69420delT | p. P23140fs | 1 | — | 1.25E-05 | Sanger | PVS1+PM2+PP4 |
| 1 | c.68101dupA | p. T22701fs | 1 | novel | NA | Sanger | PVS1+PM2+PM6+PP4 |
| 1 | c.65805delC | p. T21935fs | 1 | novel | NA | Sanger | PVS1+PM2+PM6+PP4 |
| 1 | c.65058_65061del | p. D21686fs | 1 | novel | NA | Sanger | PVS1+PM2+PM6+PP4 |
| 2 | c.G64499A | p. W21500X | 1 | novel | NA | IGV | PVS1+PM2+PM6+PP3+PP4 |
| 2 | c.64149delC | p. A21383fs | 1 | novel | NA | IGV | PVS1+PM2+PM6+PP4 |
| 1 | c.C63625T | p. R21209X | 1 | rs794729279 | 4.09E-06 | Sanger | PVS1+PM2+PP3+PP4+PP5 |
| 1 | c.C61921T | p. R20641X | 1 | rs878854324 | NA | IGV | PVS1+PM2+PP3+PP4+PP5 |
| 1 | c.61545delA | p. K20515fs | 1 | novel | NA | Sanger | PVS1+PM2+PM6+PP4 |
| 1 | c.A60829T | p. K20277X | 1 | novel | NA | IGV | PVS1+PM2+PM6+PP3+PP4 |
| 1 | c.60356_60357insGAGAT | p. I20119fs | 1 | novel | NA | IGV | PVS1+PM2+PM6+PP4 |
| 1 | c.59927-1G>A |  | 1 | novel | NA | Sanger | PVS1+PM2+PM6+PP4 |
| 1 | c.G59176T | p. E19726X | 1 | novel | NA | Sanger | PVS1+PM2+PM6+PP3+PP4 |
| 1 | c.G59062T | p. E19688X | 1 | novel | NA | Sanger | PVS1+PM2+PM6+PP3+PP4 |
| 1 | c.C57769T | p. R19257X | 1 | rs794729275 | 5.09E-06 | IGV | PVS1+PM2+PP3+PP4+PP5 |
| 1 | c.C56257T | p. Q18753X | 1 | novel | NA | Sanger | PVS1+PM2+PM6+PP3+PP4 |
| 1 | c.54811+1G>A |  | 1 | — | 4.19E-06 | IGV | PVS1+PM2+PP4 |
| 1 | c.G54622T | p. E18208X | 1 | novel | NA | Sanger | PVS1+PM2+PM6+PP3+PP4 |
| 1 | c.54470_54474del | p. K18157fs | 1 | novel | NA | Sanger | PVS1+PM2+PM6+PP4 |
| 1 | c.54413_54414insCAGA | p. E18138fs | 1 | novel | NA | Sanger | PVS1+PM2+PM6+PP4 |
| 1 | c.A54310T | p. K18104X | 1 | novel | NA | Sanger | PVS1+PM2+PM6+PP3+PP4 |
| 1 | c.54234delA | p. K18078fs | 1 | novel | NA | IGV | PVS1+PM2+PM6+PP4 |
| 1 | c.T53691G | p. Y17897X | 1 | novel | NA | Sanger | PVS1+PM2+PM6+PP4 |
| 1 | c.C53206T | p. R17736X | 1 | — | NA | Sanger | PVS1+PM2+PP3+PP4 |
| 1 | c.52405+1G>- |  | 1 | novel | NA | Sanger | PVS1+PM2+PM6+PP4 |
| 1 | c.C52021T | p. R17341X | 1 | rs926741242 | 4.33E-06 | IGV | PVS1+PM2+PP3+PP4+PP5 |
| 1 | c.51436+1G>A |  | 1 | rs761807131 | 4.09E-06 | IGV | PVS1+PP4+PP5 |
| 1 | c.G50374T | p. E16792X | 1 | novel | NA | Sanger | PVS1+PM2+PM6+PP3+PP4 |
| 1 | c.48460+2T>C |  | 1 | novel | NA | Sanger | PVS1+PM2+PM6+PP4 |
| 1 | c.C48283T | p. R16095X | 1 | rs374140736 | 4.09E-06 | IGV | PVS1+PM2+PP3+PP4+PP5 |
| 1 | c.48006_48007insAAGTA | p. L16003fs | 1 | novel | NA | IGV | PVS1+PM2+PM6+PP4 |
| 1 | c.47875+1G>A |  | 1 | rs869312047 | NA | Sanger | PVS1+PM2+PP4+PP5 |
| 1 | c.47270-1G>C |  | 1 | novel | NA | Sanger | PVS1+PM2+PM6+PP4 |
| 2 | c.44281+1G>A |  | 1 | rs771562210 | 4.08E-06 | IGV | PVS1+PM2+PP4 |
| 1 | c.C44272T | p. R14758X | 1 | rs140743001 | 1.22E-05 | IGV | PVS1+PM2+PP3+PP4+PP5 |
| 1 | c.G43854A | p. W14618X | 1 | novel | NA | IGV | PVS1+PM2+PM6+PP3+PP4 |
| 1 | c.41884+1G>T |  | 1 | novel | NA | Sanger | PVS1+PM2+PM6+PP4 |
| 1 | c.41418delA | p. K13806fs | 1 | novel | NA | Sanger | PVS1+PM2+PM6+PP4 |
| 1 | c.41032delC | p. L13678X | 1 | novel | NA | Sanger | PVS1+PM2+PM6+PP4 |
| 1 | c.13423delG | p. D4475fs | 1 | novel | NA | Sanger | PVS1+PM2+PM6+PP4 |
| 1 | c.13073delA | p. E4358fs | 1 | novel | NA | Sanger | PVS1+PM2+PM6+PP4 |
| 1 | c.11459delC | p. P3820fs | 1 | novel | NA | IGV | PVS1+PM2+PM6+PP4 |
| 1 | c.9472-1G>T |  | 1 | novel | NA | IGV | PVS1+PM2+PM6+PP4 |
| 1 | c.8902+1G>A |  | 1 | rs770392096 | 2.46E-05 | IGV | PVS1+PM2+PP4 |
| 1 | c.7698_7713del | p. N2566fs | 1 | novel | NA | IGV | PVS1+PM2+PM6+PP4 |
| 1 | c.C7501T | p. R2501X | 1 | rs781459488 | 4.07E-06 | Sanger | PVS1+PM2+PP3+PP4 |
| 1 | c.2842-1G>C |  | 1 | novel | NA | Sanger | PVS1+PM2+PM6+PP4 |
| 1 | c.G2740T | p. E914X | 0.99 | novel | NA | IGV | PVS1+PM2+PM6+PP3+PP4 |
| 1 | c.2205_2206insT | p. G736fs | 1 | novel | NA | Sanger | PVS1+PM2+PM6+PP4 |
| 1 | c.C2137T | p. R713X | 1 | rs727505277 | NA | Sanger | PVS1+PM2+PP4 |
| 1 | c.C1390T | p. Q464X | 1 | novel | NA | IGV | PVS1+PM2+PM6+PP3+PP4 |
| 1 | c.1245+2T>C |  | 1 | novel | NA | Sanger | PVS1+PM2+PM6+PP3+PP4 |
| 1 | c.G75934T | p. E25312X | 1 | novel | NA | Sanger | PVS1+PM2+PM6+PP3+PP4 |
| 2 | c.C49870T | p. R16624X | 1 | [rs1471414348](https://www.ncbi.nlm.nih.gov/SNP/snp_ref.cgi?type=rs&rs=rs1471414348) | NA | Sanger | PVS1+PM2+PM6+PP3+PP4+PP5 |
| No., indicates the number of patients; PSI, percentage of splicing in; GnomAD, indicates the global risk allele frequency in the Genome Aggregation Database; IGV, Integrative Genomics Viewer. ACMG, indicates the American College of Medical Genetics and Genomics guideline for the interpretation of sequence variants criteria. PVS, indicates evidence of pathogenicity very strong based on the ACMG criteria. PS, indicates evidence of pathogenicity strong based on the ACMG criteria. PM, indicates evidence of pathogenicity moderate based on the ACMG criteria. PP, indicates evidence supporting pathogenicity based on the ACMG criteria. | | | | | | | |

| **Table S3. Recurring tTTN in the cohort.** | | | | | |
| --- | --- | --- | --- | --- | --- |
| **Transcript** | **Coding change** | **Protein change** | **Calls (n)** | **GnomAD-EAS (n)** | ***p-* value** |
| ENST00000589042 | c.105800_105801del | p. T35267fs | 2/1041 | 0/9435 | 0.01 |
| ENST00000589042 | c.87119-1G>T |  | 2/1041 | 0/9435 | 0.01 |
| ENST00000589042 | c.86162_86163del | p. Y28721fs | 2/1041 | 0/9435 | 0.01 |
| ENST00000589042 | c.C81037T | p. R27013X | 4/1041 | 0/9435 | 0.0001 |
| ENST00000589042 | c.G64499A | p. W21500X | 2/1041 | 0/9435 | 0.01 |
| ENST00000589042 | c.64149delC | p. A21383fs | 2/1041 | 0/9435 | 0.01 |
| ENST00000589042 | c.44281+1G>A |  | 2/1041 | 0/9435 | 0.01 |

, East-Asia population of GnomAD database.

| **Table S4. Clinical Feature of DCM stratified by pathogenic missense variants (PMV) status.** | | | |
| --- | --- | --- | --- |
|  | **DCM without PMV** | **DCM with PMV** | ***p-* value** |
|  | **(n = 754)** | **(n = 287)** |  |
| **Male, n (%)** | 550 (72.9%) | 215 (74.9%) | 0.520 |
| **Age at onset (yrs)** | 51.64 ± 13.65 | 53.78 ± 13.48 | 0.024 |
| **NYHA functional class III/IV, n (%)** | 524 (69.6%) | 198 (69.0%) | 0.851 |
| **Medical history** |  |  |  |
| Hypertension, n (%) | 387 (51.3%) | 153 (53.3%) | 0.567 |
| Diabetes, n (%) | 126 (16.7%) | 53 (18.5%) | 0.508 |
| Smoking, n (%) | 314 (41.6%) | 130 (45.3%) | 0.287 |
| Stroke, n (%) | 33 (4.4%) | 13 (4.5%) | 0.921 |
| **Conduction defect/Arrhythmia** |  |  |  |
| Left bundle branch block, n (%) | 81 (10.8%) | 26 (9.1%) | 0.421 |
| Atrial fibrillation, n (%) | 167 (22.2%) | 67 (23.3%) | 0.687 |
| Non-sustained ventricular tachycardia, n (%) | 98 (13.0%) | 31 (10.8%) | 0.333 |
| Sustained ventricular tachycardia, n (%) | 6 (0.8%) | 4 (1.4%) | 0.378 |
| **Echocardiographic phenotype** |  |  |  |
| LVEDD (mm) | 65.68 ± 12.25 | 65.76 ± 10.23 | 0.927 |
| LVEDDi (mm/m^2^) | 37.20 ± 8.87 | 38.19 ± 5.52 | 0.336 |
| LVEF (%) | 30.91 (10.99%) | 31.66 (9.50%) | 0.316 |
| LAD (mm) | 44.79 ± 9.71 | 45.48 ± 9.50 | 0.300 |
| E/A ratio | 1.72 ± 1.77 | 1.72 ± 1.31 | 0.966 |
| E/e' ratio | 22.66 ± 11.52 | 24.49 ± 19.24 | 0.235 |
| LVM (g) | 295.25 ± 90.17 | 287.24 ± 94.12 | 0.218 |
| LVMi (g/m^2^) | 166.11 ± 50.43 | 169.99 ± 48.87 | 0.546 |
| **Pharmacological and device-based therapy** |  |  |  |
| Digoxin, n (%) | 356 (47.5%) | 151 (52.6%) | 0.138 |
| Diuretics, n (%) | 603 (80.4%) | 252 (87.8%) | 0.005 |
| ACEI or ARB, n (%) | 559 (74.4%) | 221 (77.0%) | 0.392 |
| Beta-blocker, n (%) | 382 (50.9%) | 149 (52.1%) | 0.723 |
| Spironolactone, n (%) | 569 (75.9%) | 229 (79.8%) | 0.179 |
| History of pacemaker implantation, n (%) | 39 (5.2%) | 14 (4.9%) | 0.840 |
| History of ICD, n (%) | 15 (2.0%) | 3 (1.0%) | 0.295 |

NYHA, New York Heart Association; LVEDD, left ventricular end-diastolic dimension; LVEDDi, left ventricular end-diastolic dimension index; LVEF, left ventricular ejection fraction; LAD, left atrial dimension; E/A ratio, early to late peak diastolic mitral flow velocity ratio; E/e’ ratio, ratio of early peak diastolic mitral velocity/peak early diastolic mitral annular velocity; LVM, left ventricular mass; LVMi, left ventricular mass index; ACEI, angiotensin converting enzyme inhibitor; ARB, angiotensin receptor blockers; ICD, Implantable cardioverter defibrillator.

**Table S5. Cardiac arrhythmia and conduction defects in tTTN stratified by variant type.**

|  | Nonsense | Frameshift | splice site | *p*-value |
| --- | --- | --- | --- | --- |
|  | n = 55 | n = 48 | n = 17 |  |
| **Arrhythmia** |  |  |  |  |
| Left bundle branch block, n (%) | 2 (3.6%) | 0 (0.0%) | 2 (4.2%) | 0.703 |
| Atrial fibrillation, n (%) | 9 (16.4%) | 6 (35.3%) | 16 (33.3%) | 0.092 |
| Non-sustained ventricular tachycardia, n (%) | 7 (12.7%) | 2 (11.8%) | 9 (18.8%) | 0.640 |
| Sustained ventricular tachycardia, n (%) | 1 (1.8%) | 1 (5.9%) | 1 (2.1%) | 0.626 |

**Table S6. Clinical Feature of DCM patients with tTTN Stratified by sex.**

|  | **Female** | **Male** | ***p* value** |
| --- | --- | --- | --- |
|  | **(n = 22)** | **(n = 98)** |  |
| **Age at onset (yrs)** | 53.32 ± 10.62 | 49.81 ± 13.14 | 0.245 |
| **NYHA functional class III/IV, n (%)** | 19 (86.4%) | 75 (76.5%) | 0.468 |
| **Conduction defect/Arrhythmia** |  |  |  |
| Left bundle branch block, n (%) | 0 (0.0%) | 4 (4.1%) | 0.759 |
| Atrial fibrillation, n (%) | 9 (40.9%) | 22 (22.4%) | 0.129 |
| Non-sustained ventricular tachycardia, n (%) | 3 (13.6%) | 15 (15.3%) | 0.889 |
| Sustained ventricular tachycardia, n (%) | 0 (0.0%) | 3 (3.1%) | 0.940 |
| **Echocardiographic phenotype** |  |  |  |
| LVEDDi (mm/g/m^2^)) | 41.11 ± 6.95 | 36.45 ± 4.93 | 0.036 |
| LVEF (%) | 31.55 ± 9.29 | 28.28 ± 8.52 | 0.114 |
| LVMi (g/m^2^) | 209.64 ± 73.32 | 136.09 ± 30.96 | <0.001 |
| E/A ratio | 1.32 ± 0.61 | 1.62 ± 0.88 | 0.261 |
| E/e' ratio | 19.58 ± 5.39 | 21.61 ± 12.25 | 0.600 |
| LAD (mm) | 44.7 ± 8.0 | 46.0 ± 8.7 | 0.502 |

NYHA, New York Heart Association; LVEDDi, left ventricular end-diastolic dimension index; LVEF, left ventricular ejection fraction; LVMi, left ventricular mass index; E/A ratio, early to late peak diastolic mitral flow velocity ratio; E/e’ ratio, ratio of early peak diastolic mitral velocity/peak early diastolic mitral annular velocity; LAD, left atrial dimension.

**Table S7. Results of univariable Cox Proportional hazard modelling predicting end-point.**

| **Variable** | **Primary end-point** | | ***P* value** | **Second end-point** | | ***P* value** |
| --- | --- | --- | --- | --- | --- | --- |
|  | **HR** | **95% CI** |  | **HR** | **95% CI** |  |
| Gender | 0.863 | 0.695 - 1.072 | 0.182 | 0.801 | 0.661 - 0.970 | 0.023 |
| Age at onset | 1.016 | 1.009 - 1.024 | < 0.0001 | 1.018 | 1.012 - 1.025 | < 0.0001 |
| Hypertension | 1.032 | 0.848 - 1.255 | 0.754 | 1.147 | 0.962 - 1.368 | 0.126 |
| Dyslipidemia | 0.870 | 0.625 - 1.210 | 0.408 | 0.954 | 0.714 - 1.275 | 0.752 |
| Diabetes | 1.333 | 1.046 - 1.698 | 0.020 | 1.377 | 1.109 - 1.71 | 0.004 |
| Stroke | 1.551 | 1.035 - 2.326 | 0.034 | 1.484 | 1.026 - 2.147 | 0.036 |
| NYHA functional class III/IV | 2.238 | 1.739 - 2.879 | < 0.0001 | 1.955 | 1.575 - 2.425 | < 0.0001 |
| Left bundle branch block | 1.149 | 0.855 - 1.546 | 0.358 | 1.280 | 0.986 - 1.66 | 0.064 |
| Atrial fibrillation | 1.556 | 1.252 - 1.934 | < 0.0001 | 1.467 | 1.204 - 1.786 | < 0.0001 |
| Non-sustained ventricular tachycardia | 1.056 | 0.785 - 1.420 | 0.721 | 1.254 | 0.972 - 1.617 | 0.081 |
| Sustained ventricular tachycardia | 0.613 | 0.196 - 1.910 | 0.398 | 0.712 | 0.266 - 1.906 | 0.498 |
| E/A ratio | 0.976 | 0.890 - 1.072 | 0.614 | 0.964 | 0.884 - 1.050 | 0.399 |
| E/e' ratio | 0.999 | 0.988 - 1.011 | 0.892 | 0.997 | 0.987 - 1.008 | 0.623 |
| LVMi | 1.003 | 0.999 - 1.008 | 0.178 | 1.004 | 1.000 - 1.009 | 0.033 |
| LVEDDi | 1.083 | 1.051 - 1.117 | < 0.0001 | 1.068 | 1.039 - 1.097 | < 0.0001 |
| LAD | 1.354 | 1.204 - 1.522 | < 0.0001 | 1.303 | 1.173 - 1.448 | < 0.0001 |
| LVEF | 0.978 | 0.968 - 0.989 | < 0.0001 | 0.984 | 0.975 - 0.993 | 0.001 |
| Digoxin | 1.129 | 0.929 - 1.372 | 0.224 | 1.207 | 1.013 - 1.438 | 0.036 |
| Diuretics | 0.906 | 0.704 - 1.166 | 0.443 | 1.019 | 0.808 - 1.286 | 0.872 |
| ACEI or ARB | 0.537 | 0.436 - 0.661 | < 0.0001 | 0.607 | 0.502 - 0.734 | < 0.0001 |
| Beta-blocker | 0.494 | 0.404 - 0.604 | < 0.0001 | 0.551 | 0.461 - 0.658 | < 0.0001 |
| Spironolactone | 0.728 | 0.585 - 0.906 | 0.004 | 0.785 | 0.643 - 0.959 | 0.018 |
| History of pacemaker implantation | 1.068 | 0.689 - 1.658 | 0.768 | 1.042 | 0.702 - 1.546 | 0.838 |
| History of ICD | 0.909 | 0.406 - 2.037 | 0.817 | 0.819 | 0.388 - 1.727 | 0.600 |

| **Table S8. The list of pathogenic variants in the other causal genes of DCM.** | | | | | | | |
| --- | --- | --- | --- | --- | --- | --- | --- |
| Gene | **No.** | **Transcript** | **Coding change** | **Protein change** | **Novelty** | **Confirmed** | **ACMG** |
| ACTN2 | 0 | - | - | - |  | - | - |
| LMNA | 4 | LMNA: NM_001282625 | c.C736T | p. Q246X | rs267607587 | IGV | PVS1+PM2+PP3 |
|  |  | LMNA: NM_001282625 | c.21_22del | p. R7fs | Novel | IGV | PVS1+PM2 |
|  |  | LMNA: NM_001282625 | c.1489-1->ATCT | - | Novel | Sanger | PVS1+PM2 |
|  |  | LMNA: NM_001257374 | c.1626dupA | p. R542fs | Novel | Sanger | PVS1+PM2 |
| NEXN | 0 | - | - | - |  | - | - |
| TNNT2 | 9 | TNNT2: NM_001276345 | c.659_661del | p. 220_221del | rs121964859 | Sanger | PS4+PM2++PM4+PP1 |
|  |  | TNNT2: NM_001276345 | c.G452A | p. R151Q | rs730881101 | IGV | PS4+PM2+PP3+PP5 |
|  |  | TNNT2: NM_001276345 | c.C304T | p. R102W | rs397516456 | Sanger | PS3+PM2+PP3+PP5 |
| DES | 0 | - | - | - |  | - | - |
| CAV3 | 0 | - | - | - |  | - | - |
| SCN5A | 2 | SCN5A: NM_000335 | c.G4054A | p. V1352M | rs199473233 | Sanger | PM1+PM2+PP3+PP5 |
|  |  | SCN5A: NM_000335 | c.G1993T | p. A665S | rs756474485 | Sanger | PM1+PM2+PP3+PP5 |
| TNNC1 | 0 | - | - | - |  | - | - |
| PDLIM3 | 1 | PDLIM3: NM_014476 | c.906-2A>G | - | Novel | Sanger | PVS1+PM2 |
| SDHA | 1 | SDHA: NM_001330758 | c.826delG | p. G276fs | Novel | Sanger | PVS1+PM2 |
| SGCD | 0 | - | - | - |  | - | - |
| DSP | 8 | DSP: NM_004415 | c.C748T | p. Q250X | Novel | IGV | PVS1+PM2+PP3 |
|  |  | DSP: NM_004415 | c.C2821T | p. R941X | rs730880082 | IGV | PVS1+PM2+PP3+PP5 |
|  |  | DSP: NM_004415 | c.C4198T | p. R1400X | rs770873593 | Sanger | PVS1+PM2+PP5 |
|  |  | DSP: NM_004415 | c.1224_1227del | p. N408fs | Novel | IGV | PVS1+PM2 |
|  |  | DSP: NM_004415 | c.5016delG | p. Q1672fs | Novel | Sanger | PVS1+PM2 |
|  |  | DSP: NM_004415 | c.7047_7048del | p. S2349fs | Novel | IGV | PVS1+PM2 |
|  |  | DSP: NM_004415 | c.7875_7876del | p. T2625fs | Novel | Sanger | PVS1+PM2 |
| LAMA4 | 0 | - | - | - |  | - | - |
| PLN | 2 | PLN: NM_002667 | c.C25T | p. R9C | rs111033559 | IGV | PS4+PM2+PP3+PP5 |
| SYNE1 | 4 | SYNE1: NM_033071 | c.C2827T | p. R943X | Novel | Sanger | PVS1+PM2+PP3 |
|  |  | SYNE1: NM_033071 | c.T1124A | p. L375X | Novel | Sanger | PVS1+PM2+PP3 |
|  |  | SYNE1: NM_033071 | c.16024-2->T | - | rs752661566 | Sanger | PVS1+PM2 |
|  |  | SYNE1: NM_033071 | c.10880_10881del | p. K3627fs | Novel | IGV | PVS1+PM2 |
| FKTN | 0 | - | - | - |  | - | - |
| FXN | 0 | - | - | - |  | - | - |
| ANKRD1 | 2 | ANKRD1NM_014391 | c.222dupA | p. L75fs | rs776659587 | Sanger | PVS1+PM2 |
| BAG3 | 3 | BAG3: NM_004281 | c.C367T | p. R123X | rs387906875 | Sanger | PVS1+PP3+PP5 |
|  |  | BAG3: NM_004281 | c.66delG | p. L22fs | Novel | IGV | PVS1+PM2 |
|  |  | BAG3: NM_004281 | c.112_113insA | p. F38fs | Novel | Sanger | PVS1+PM2 |
| LDB3 | 0 | - | - | - |  | - | - |
| RBM20 | 4 | RBM20: NM_001134363 | c.C532T | p. R178X | rs794729161 | Sanger | PVS1+PM2+PP3 |
|  |  | RBM20: NM_001134363 | c.C2062T | p. R688X | rs794729150 | IGV | PVS1+PM2 |
|  |  | RBM20: NM_001134363 | c.118delG | p. G40fs | Novel | IGV | PVS1+PM2 |
|  |  | RBM20: NM_001134363 | c.2687delA | p. E896fs | Novel | Sanger | PVS1+PM2 |
| VCL | 0 | - | - | - |  | - | - |
| CRYAB | 1 | CRYAB: NM_001289807 | c.G3A | p. M1I | rs397516686 | Sanger | PM1+PM2+PP3+PP5 |
| CSRP3 | 1 | CSRP3: NM_003476 | c.C364T | p. R122X | rs902082118 | Sanger | PVS1+PM2+PP3+PP5 |
| ILK | 0 | - | - | - |  | - | - |
| MYBPC3 | 10 | MYBPC3: NM_000256 | c.3624delC | p. P1208fs | rs397516030 | Sanger | PVS1+PM2 |
|  |  | MYBPC3: NM_000256 | c.G3A | p. M1I | rs397516045 | Sanger | PS1+PM2+PP5 |
|  |  | MYBPC3: NM_000256 | c.C3811T | p. R1271X | rs397516042 | Sanger | PVS1+PS3+PM2+PP3+PP5 |
|  |  | MYBPC3: NM_000256 | c.T571C | p. W191R | rs730880622 | IGV | PM1+PM2+PP3+PP5 |
|  |  | MYBPC3: NM_000256 | c.3049_3050insC | p. E1017fs | Novel | Sanger | PVS1+PM2 |
|  |  | MYBPC3: NM_000256 | c.3033delG | p. G1011fs | Novel | IGV | PVS1+PM2 |
|  |  | MYBPC3: NM_000256 | c.2149-2->C | - | rs748128261 | IGV | PVS1+PM2 |
| ABCC9 | 0 | - | - | - |  | - | - |
| MYH6 | 2 | MYH6: NM_002471 | c.5164-1G>T | - | Novel | Sanger | PVS1+PM2 |
|  |  | MYH6: NM_002471 | c.2686-1G>A | - | Novel | IGV | PVS1+PM2 |
| MYH7 | 21 | MYH7: NM_000257 | c.G5704C | p. E1902Q | rs187073962 | IGV | PM1+PM2+PP2+PP3 |
|  |  | MYH7: NM_000257 | c.C5689T | p. R1897C | rs756655803 | Sanger | PM1+PM2+PP2+PP3 |
|  |  | MYH7: NM_000257 | c.G4589A | p. R1530Q | rs730880803 | Sanger | PM1+PM2+PP2+PP3 |
|  |  | MYH7: NM_000257 | c.C4441T | p. L1481F | rs767666398 | Sanger | PM1+PM2+PP2+PP3 |
|  |  | MYH7: NM_000257 | c.G4348A | p. D1450N | rs397516211 | IGV | PM1+PM2+PP2+PP3 |
|  |  | MYH7: NM_000257 | c.G3803A | p. R1268H | rs397516194 | IGV | PM1+PM2+PP2+PP3 |
|  |  | MYH7: NM_000257 | c.C3235T | p. R1079W | rs192722540 | Sanger | PM1+PM2+PP2+PP3 |
|  |  | MYH7: NM_000257 | c.A2981G | p. K994R | rs1298804415 | IGV | PM1+PM2+PP2+PP3 |
|  |  | MYH7: NM_000257 | c.G1681A | p. A561T | rs730880878 | Sanger | PM1+PM2+PP2+PP5 |
|  |  | MYH7: NM_000257 | c.C1322T | p. T441M | rs121913653 | IGV | PM1+PM2+PP2+PP5 |
|  |  | MYH7: NM_000257 | .C391G | p. L131V | Novel | Sanger | PM1+PM2+PP2+PP3 |
|  |  | MYH7: NM_000257 | c.C4498T | p. R1500W | rs45544633 | IGV | PM1+PM2+PP2+PP5 |
|  |  | MYH7: NM_000257 | c.C1357T | p. R453C | rs121913625 | Sanger | PM1+PM2+PP2+PP3+PP5 |
| ACTC1 | 0 | - | - | - |  | - | - |
| TPM1 | 1 | TPM1: NM_00101800 | c.C679T | p. Q227X | Novel | Sanger | PVS1+PM2+PP3 |
| TCAP | 0 | - | - | - |  | - | - |
| DSG2 | 5 | DSG2: NM_001943 | c.A593G | p. Y198C | rs786204291 | IGV | PM1+PM2+PP3+PP5 |
|  |  | DSG2: NM_001943 | c.T710A | p. L237X | Novel | Sanger | PVS1+PM2+PP3 |
|  |  | DSG2: NM_001943 | c.1681delA | p. K561fs | Novel | Sanger | PVS1+PM2 |
| TNNI3 | 0 | - | - | - |  | - | - |
| DMD | 0 | - | - | - |  | - | - |
| LAMP2 | 2 | LAMP2: NM_001122606 | c.928+1G>A | - | Novel | Sanger | PVS1+PM2 |

No., indicates the number of patients; PSI, percentage of splicing in; GnomAD, indicates the global risk allele frequency in the Genome Aggregation Database; ACMG, indicates the American College of Medical Genetics and Genomics guideline for the interpretation of sequence variants criteria. PVS, indicates evidence of pathogenicity very strong based on the ACMG criteria. PS, indicates evidence of pathogenicity strong based on the ACMG criteria. PM, indicates evidence of pathogenicity moderate based on the ACMG criteria. PP, indicates evidence supporting pathogenicity based on the ACMG criteria.

**Table S9. Phenotype in those with tTTN vs those with tTTN and pathogenic variants in OCG.**

|  | **tTTN+/OCG-** | **tTTN+/OCG+** | *p*-value |
| --- | --- | --- | --- |
|  | (n = 112) | (n = 8) |  |
| **Age at onset (yrs)** | 50.06 ± 12.70 | 55.88 ± 12.99 | 0.214 |
| **NYHA functional class III/IV, n (%)** | 87 (77.7%) | 7 (87.5%) | 0.836 |
| **Conduction defect/Arrhythmia** |  |  |  |
| Left bundle branch block, n (%) | 3 (2.7%) | 1 (12.5%) | 0.634 |
| Atrial fibrillation, n (%) | 30 (26.8%) | 1 (12.5%) | 0.636 |
| Non-sustained ventricular tachycardia, n (%) | 16 (14.3%) | 2 (25.0%) | 0.758 |
| Sustained ventricular tachycardia, n (%) | 3 (2.7%) | 0 (0.0%) | 0.982 |
| **Echocardiographic phenotype** |  |  |  |
| LVEDD (mm) | 66.0 ± 6.9 | 64.0 ± 5.7 | 0.423 |
| LVEDDi (mm/m^2^) | 37.40 ± 5.72 | 38.60 ± NA | NA |
| LVEF (%) | 28.76 ± 8.89 | 30.62 ± 6.05 | 0.562 |
| LAD (mm) | 46.1 ± 8.7 | 41.5 ± 4.0 | 0.141 |
| E/A ratio | 1.54 ± 0.85 | 1.89 ± 0.72 | 0.336 |
| E/e' ratio | 21.22 ± 11.05 | 18.10 ± 4.10 | 0.696 |
| LVM (g) | 277.03 ± 64.93 | 269.68 ± 40.68 | 0.753 |
| LVMi (g/m^2^) | 151.81 ± 52.58 | 142.85 ± NA | NA |
| **History of pacemaker implantation, n (%)** | 1 (0.9%) | 0 (0.0%) | 0.993 |
| **History of ICD, n (%)** | 2 (1.8%) | 0 (0.0%) | 0.989 |

NYHA, New York Heart Association; LVEDD, left ventricular end-diastolic dimension; LVEDDi, left ventricular end-diastolic dimension index; LVEF, left ventricular ejection fraction; LAD, left atrial dimension; LVM, left ventricular mass; LVMi, left ventricular mass index; ICD, Implantable cardioverter defibrillator.

**Table S10. Clinical Feature of DCM Stratified by pathogenic variants status.**

|  | **OCG+/tTTN-** | **tTTN+/OCG-** | ***p*-value** |
| --- | --- | --- | --- |
|  | (n = 71) | (n = 112) |  |
| **Age at onset (yrs)** | 50.72 (12.28) | 50.06 (12.70) | 0.731 |
| **NYHA functional class III/IV, n (%)** | 50 ± 70.4 | 87 ± 77.7 | 0.354 |
| **Conduction defect/Arrhythmia** |  |  |  |
| Left bundle branch block, n (%) | 8 (11.3%) | 3 (2.7%) | 0.039 |
| Atrial fibrillation, n (%) | 11 (15.5%) | 30 (26.8%) | 0.109 |
| Non-sustained ventricular tachycardia, n (%) | 8 (11.3%) | 16 (14.3%) | 0.715 |
| Sustained ventricular tachycardia, n (%) | 1 (1.4%) | 3 (2.7%) | 0.957 |
| **Echocardiographic phenotype** |  |  |  |
| LVEDD (mm) | 6.76 ± 0.74 | 6.60 ± 0.69 | 0.146 |
| LVEDDi (mm/m^2^) | 37.53 ± 5.35 | 37.40 ± 5.72 | 0.938 |
| LVEF (%) | 33.07 ± 10.59 | 28.76 ± 8.89 | 0.004 |
| LAD (mm) | 4.67 ± 0.88 | 4.61 ± 0.87 | 0.658 |
| E/A ratio | 1.92 ± 1.76 | 1.54 ± 0.85 | 0.142 |
| E/e' ratio | 23.48 ± 11.74 | 21.22 ± 11.05 | 0.429 |
| LVM (g) | 305.49 ± 83.78 | 277.03 ± 64.93 | 0.014 |
| LVMi (g/m^2^) | 148.98 ± 36.02 | 151.81 ± 52.58 | 0.834 |
| **History of pacemaker implantation, n (%)** | 7 (9.9%) | 1 (0.9%) | 0.012 |
| **History of ICD, n (%)** | 2 (2.8%) | 2 (1.8%) | 0.972 |

NYHA, New York Heart Association; LVEDD, left ventricular end-diastolic dimension; LVEDDi, left ventricular end-diastolic dimension index; LVEF, left ventricular ejection fraction; LAD, left atrial dimension; LVM, left ventricular mass; LVMi, left ventricular mass index; ICD, Implantable cardioverter defibrillator.
